# Sputum Exosomal microRNAs as Non-Invasive Biomarkers in COPD: A Cross-Sectional Study

**DOI:** 10.3390/biomedicines13123027

**Published:** 2025-12-10

**Authors:** Ourania S. Kotsiou, Irene Tsilioni, Aikaterini Tsingene, Aikaterini Katsanaki, Nikolaos A. A. Balatsos, Erasmia Rouka, Zoe Daniil, Konstantinos I. Gourgoulianis

**Affiliations:** 1 Laboratory of Human Pathophysiology, Department of Nursing, University of Thessaly, 41500 Larissa, Greece; 2 Department of Immunology, Tufts University School of Medicine, Boston, MA 02111, USA; 3 Department of Respiratory Medicine, University of Thessaly, 41100 Larissa, Greece; 4 Department of Biochemistry, University of Thessaly, 41500 Larissa, Greecekakats1992@yahoo.gr (A.K.);; 5 Department of Nursing, University of Thessaly, 41500 Larissa, Greece; errouka@uth.gr

**Keywords:** chronic obstructive pulmonary disease, exosomes, microRNA, sputum biomarkers

## Abstract

**Background:** Chronic obstructive pulmonary disease (COPD) is a heterogeneous condition marked by airway inflammation, airflow limitation, and structural remodeling. Exosomal microRNAs (exo-miRNAs) are stable, cell-free biomarkers reflecting airway molecular changes. While serum and BALF exosomal miRNAs have been examined, sputum-derived profiles remain underexplored. **Methods:** Induced sputum was collected from 20 clinically stable COPD patients and 10 age-matched healthy controls. Exosomes were isolated by polymer-based precipitation and verified by transmission electron microscopy and Western blotting for CD9 and CD81. Nine candidate miRNAs (miR-21, miR-155, miR-34a, miR-126, miR-210, miR-146a, miR-199a-5p, miR-223, miR-1246) were quantified by RT-qPCR. Group comparisons used the Mann–Whitney U test, correlations Pearson’s r, and diagnostic accuracy ROC analysis. **Results:** Sputum-derived exosomes displayed characteristic morphology and canonical protein markers. COPD patients showed significant dysregulation of exosomal miRNAs, including upregulation of miR-21 (fold change = 3.4; 95% CI: 0.12–0.64 vs. 0.18–0.22; *p* < 0.001) and miR-223 (fold change = 2.1; 95% CI: 0.00–3.79 vs. 0.86–1.22; *p* = 0.004), and downregulation of miR-155 (fold change = 0.35; 95% CI: 0.43–0.67 vs. 0.86–1.22; *p* = 0.002), miR-126 (fold change = 0.42; 95% CI: 0.30–0.39 vs. 0.80–1.42; *p* = 0.009), and miR-146a (fold change = 0.28; 95% CI: 0.49–1.12 vs. 0.87–1.35; *p* = 0.006). miR-21 correlated with symptom burden (CAT; r = 0.445; *p* = 0.049). Among individual biomarkers, miR-155 exhibited the best diagnostic performance for COPD detection (AUC = 0.730; 95% CI: 0.53–0.93), which further improved when combined with miR-126 and miR-146a (AUC = 0.841; 95% CI: 0.69–0.98). For disease stratification, miR-126 most effectively discriminated mild from moderate-to-severe COPD (AUC = 0.728; 95% CI: 0.50–0.96). These results indicate that sputum-derived exosomal miRNAs—particularly miR-155, miR-126, and miR-146a—may serve as promising non-invasive biomarkers for COPD diagnosis and clinical phenotyping. **Conclusions:** Sputum exosomal miRNAs reveal a distinct COPD-specific signature reflecting inflammation, impaired repair, and immune dysregulation. Composite panels incorporating miR-155, miR-126, and miR-146a enhance diagnostic accuracy and could be integrated into non-invasive workflows for COPD detection and staging.

## 1. Introduction

Chronic obstructive pulmonary disease (COPD) is a leading cause of morbidity and mortality worldwide, characterized by persistent airway inflammation, progressive and largely irreversible airflow limitation, and recurrent bacterial colonization of the lower respiratory tract [1]. Despite advances in both pharmacological and non-pharmacological therapies, COPD remains a heterogeneous disease, with distinct phenotypes and variable trajectories of disease progression. Identifying non-invasive biomarkers that accurately reflect the airway microenvironment is a critical step toward precision medicine approaches for phenotypic stratification and therapeutic monitoring [2].

Extracellular vesicles (EVs), including exosomes (30–150 nm), have emerged as promising biomarker reservoirs because they carry stable cargos of proteins, lipids, and nucleic acids that reflect the physiological and pathological state of their parent cells [3,4]. Exosomes are particularly enriched in microRNAs (miRNAs), molecules protected from enzymatic degradation that play key roles in intercellular communication. In respiratory diseases, miRNA-containing exosomes have been implicated as both diagnostic tools and potential therapeutic vectors [5]. For example, in preclinical lung models, exosomal miR-155 and miR-146a reciprocally regulate inflammatory signaling pathways, while miR-21, let-7, and the miR-200 and miR-290 families contribute to epithelial repair and immunomodulation [6,7,8,9].

In COPD, altered EV and exosome biology has been reported in tissues, blood, bronchoalveolar lavage fluid (BALF), and sputum, implicating aberrant vesicle cargo in processes such as epithelial–mesenchymal transition, immune dysregulation, and airway remodeling [10,11,12]. Our recent study provided the first detailed characterization of sputum-derived EVs in COPD, demonstrating a two-fold enrichment of 150–200 nm vesicles and identifying a protein concentration cut-off of 55 µg·mL^−1^ that discriminates COPD from healthy subjects with 80% sensitivity and 70% specificity [13]. These data suggest that sputum EVs represent an accessible and disease-relevant compartment for biomarker discovery. However, the miRNA cargo of sputum exosomes in COPD remains underexplored, even though sputum is a simple, minimally invasive biofluid that directly reflects the lower-airway milieu.

Previous studies have shown that several exosomal miRNAs—such as miR-21, miR-155 and miR-126—are dysregulated in COPD, where they contribute to hypoxia responses, epithelial injury, and chronic inflammation [10,11,12,14,15,16]. However, most evidence comes from tissue biopsies, serum or BALF, biological sources that may not accurately reflect airway-level molecular changes. In contrast, sputum-derived exosomes originate directly from the lower airways and may therefore provide a more disease-relevant matrix for understanding local pathophysiology. Despite this advantage, systematic profiling of sputum exosomal miRNAs in COPD remains limited. Addressing this gap is critical for clarifying compartment-specific miRNA regulation and for defining the value of airway-derived exosomal miRNAs as minimally invasive diagnostic or mechanistic biomarkers. Building on our preliminary observations, the present study quantifies selected immune- and hypoxia-related miRNAs in sputum-derived exosomes from COPD patients versus healthy controls, and evaluates their mechanistic relevance and diagnostic potential in COPD.

## 2. Materials and Methods

### 2.1. Study Population

We recruited 20 consecutive outpatients with clinically stable COPD, volunteers who attended the Respiratory Medicine Department of the University of Thessaly between January and March 2025.

A formal a priori power calculation was not feasible because established effect sizes for sputum-derived exosomal miRNAs in COPD are not available in the literature [2,16]. Therefore, the sample size (20 COPD and 10 controls) was determined pragmatically based on feasibility constraints, previous comparable exosomal miRNA studies in COPD, and the exploratory nature of this work. The study was designed as a hypothesis-generating analysis consistent with established practice in early-stage biomarker discovery.

In addition to demographic data, detailed clinical characteristics were recorded, including smoking history, comorbidities (arterial hypertension, hyperlipidemia, diabetes mellitus, coronary artery disease, heart failure, chronic kidney disease, depression), as well as ongoing treatments. COPD therapy—such as long-acting bronchodilators (LABA, LAMA), inhaled corticosteroids (ICS), ICS/LABA combinations, and triple therapy—together with medications used for comorbid conditions (including antihypertensives, statins, antidiabetic agents, and antidepressants) were documented for all participants.

Inclusion criteria required participants to be adults aged 40 years or older, with a confirmed diagnosis of COPD according to GOLD criteria, defined by a post-bronchodilator FEV_1_/FVC ratio below 0.70 [1]. All COPD participants were clinically stable, without any exacerbation, respiratory infection, or systemic corticosteroid use within the preceding eight weeks, and were able to produce adequate induced sputum samples for exosomal analysis.

Exclusion criteria were designed to minimize potential confounders known to influence exosomal miRNA expression. Individuals were excluded if they had smoked actively within the last six months or had a diagnosis of asthma, bronchiectasis, interstitial lung disease, cystic fibrosis, lung cancer, or any other chronic lung disease. Additional exclusions included systemic inflammatory, autoimmune, or rheumatologic disorders; chronic infections such as tuberculosis; recent pneumonia or acute respiratory illness; and any malignancy within the previous five years. Patients receiving chronic systemic corticosteroid therapy or other immunosuppressive medications were also excluded.

To ensure clinical stability, individuals with uncontrolled diabetes mellitus or unstable cardiovascular disease, as well as pregnant or breastfeeding women, were not eligible for participation. These refinements provide a clearer description of the study population and ensure that conditions known to alter immune or exosomal pathways were minimized, thereby strengthening the interpretability of the miRNA profiles observed in COPD.

Ten age-matched volunteers without respiratory disease served as the control group. All participants provided informed written consent; the study was approved by the Institutional Ethics Committee (Approval No. 2024-12-012) and was granted by the Hellenic Thoracic Society (Funding Number: 3/22). To minimize selection bias, consecutive eligible patients were recruited, and laboratory analyses were performed blinded to group allocation.

### 2.2. Sample Collection and Processing

Sputum was induced by nebulizing hypertonic saline (4.5%). Samples were processed within 2 h using dithiothreitol, filtered, and centrifuged (300× *g*, 10 min) to remove cells and debris, following protocols previously established by our team [13].

### 2.3. Exosome Isolation and Characterization

Cell-free supernatants were subjected to polymer-based precipitation using Total Exosome Isolation™ Reagent (Invitrogen, Carlsbad, CA, USA) and incubated overnight at 4 °C. The resulting samples were centrifuged at 10,000× *g* for 1 h and the exosome pellets were resuspended in PBS [13]. Exosomal morphology was visualized by TEM (JEOL JEM-1400, JEOL Ltd., Tokyo, Japan); protein concentration was determined by BCA assay (Thermo Fisher Scientific, Waltham, MA, USA). Western blotting detected exosomal markers CD9 and CD81 (primary antibodies 1:1000).

### 2.4. RNA Extraction and miRNA Quantification

Exosomal RNA was isolated using the miRNeasy^®^ Micro Kit (Qiagen, Hilden, Germany). RNA purity and concentration were assessed using NanoDrop spectrophotometry (Thermo Fisher Scientific, Waltham, MA, USA). All samples met acceptable quality thresholds (A260/A280: 1.8–2.1; A260/A230 > 1.8) prior to downstream processing. U6 was selected as the endogenous control after confirming its stable expression across all sputum-derived exosomal samples. We acknowledge that the use of a single endogenous control without exogenous spike-in controls may limit normalization accuracy, and this is discussed in the limitations section. Reverse transcription was performed with the TaqMan™ Advanced miRNA cDNA Synthesis Kit (Thermo Fisher Scientific, Waltham, MA, USA). Quantitative PCR (qPCR) was carried out on a QuantStudio 5 system with TaqMan™ Fast Advanced Master Mix (Thermo Fisher Scientific, Waltham, MA, USA).

### 2.5. Panel Selection by Bioinformatic Analyses in COPD

Candidate miRNAs were selected following bioinformatic analyses relative to disease association and biological pathway annotations, as well as key words (inflammation, infection), using the miRBase [17] and miRPathDB 2.0 [18] tools, respectively, prioritizing molecules with confirmed COPD association as evident by previously published studies. The final qPCR panel comprised: miR-21-5p (000397), miR-155-5p (002623), miR-34a-5p (000426), miR-126-3p (002228), miR-210-3p (000512), miR-146a-5p (000468), miR-199a-5p (000499), miR-223-3p (002295), miR-1246 (462580), and U6 (001973) as the endogenous control. Each reaction was run in triplicate.

### 2.6. Statistical Analysis

Data were analyzed using GraphPad Prism 10 (GraphPad Software, San Diego, CA, USA). Continuous variables are presented as median (IQR). Normality was assessed using the Shapiro–Wilk test, and group comparisons were performed with the Mann–Whitney U test. Fold-change values were calculated as 2^−ΔΔCt^. All hypothesis tests were two-sided, with statistical significance set at *p* < 0.05.

Diagnostic performance was assessed using receiver operating characteristic (ROC) curves. For each ROC analysis, *p*-values were calculated under the null hypothesis that the true AUC equals 0.5, indicating no discriminative ability. AUC estimates are reported together with their 95% confidence intervals and associated *p*-values. Pairwise comparisons between AUCs were performed using the two-sided DeLong test, also with α = 0.05.

For correlation analyses, raw Ct values were converted to 2^−ΔCt^ values and subsequently log-transformed to improve distribution symmetry. Pearson’s r coefficients were calculated, and potential outliers were examined using boxplots and leverage statistics.

## 3. Results

### 3.1. Participant Characteristics

The COPD and control cohorts were comparable in sex distribution (χ^2^ = 0.02, *p* = 0.89). Among the COPD subjects, 90% were former smokers with a median smoking history of 40 pack-years, whereas the control group included both never-smokers and former light smokers, all of whom had normal age-adjusted spirometric values and no respiratory symptoms. The clinical characteristics of the study participants are presented in Table 1.

No participants in either group had chronic kidney disease, autoimmune or systemic inflammatory diseases, cancer, or recent infections. Most COPD patients were receiving guideline-based inhaled therapy (LAMA: 70%; LABA: 65%; ICS: 55%; triple therapy: 40%). With respect to medications for comorbid conditions, antihypertensive and lipid-lowering drugs were used more frequently among COPD patients, consistent with the higher prevalence of arterial hypertension (90% vs. 50%, *p* = 0.140) and hyperlipidemia (70% vs. 50%, *p* = 0.240), although these differences were not statistically significant. Antidiabetic therapy was more common in COPD patients (20% vs. 10%, *p* = 0.640), and use of antiplatelet or anti-ischemic agents paralleled the slightly higher prevalence of coronary artery disease (15% vs. 10%, *p* = 0.990).

The slightly lower mean FEV_1_/FVC ratio observed in the control group (76 ± 2) is attributable to the inclusion of several former smokers without respiratory symptoms and with normal age-adjusted spirometric values. Prior smoking exposure is known to cause small reductions in FEV_1_/FVC even in otherwise healthy individuals, which explains this finding.

### 3.2. Exosome Verification

Transmission Electron Microscopy (TEM) confirmed the presence of typical cup-shaped vesicles. Western blotting showed strong bands for the exosomal markers CD9 and CD81, confirming exosome enrichment, as previously reported [13]. Representative TEM images of exosomes and Western blot bands for CD9 and CD81 have now been added as Supplementary Figure S1.

### 3.3. Differential miRNA Expression

**Figure 1:**
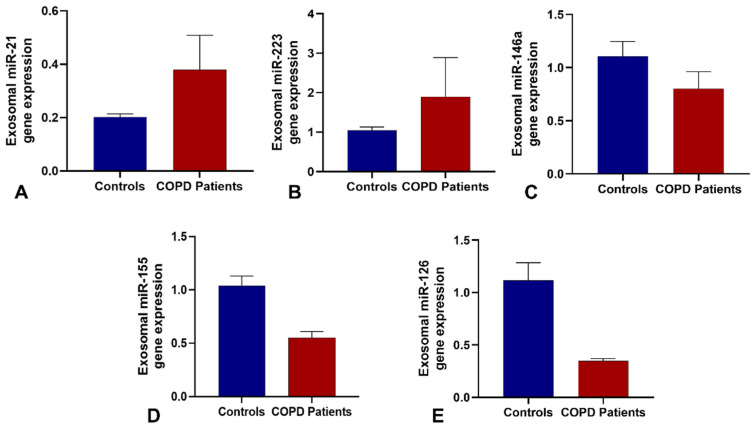
Differential expression of selected sputum-derived exosomal microRNAs between study groups. Expression levels of (**A**) miR-21; (**B**) miR-223; (**C**) miR-146a; (**D**) miR-155; and (**E**) miR-126 in sputum-derived exosomes from healthy controls and COPD patients. Note: Bars represent mean fold-change (±standard error) relative to healthy controls. Blue bars indicate healthy controls; red bars indicate COPD patients. Positive values denote up-regulation; negative values denote down-regulation compared to control group. miRNA targets were pre-selected after bioinformatic analyses. Statistical significance was determined using the Mann–Whitney U test (*p* < 0.05).

Relative expression levels are shown in Figure 1. miR-21 and miR-223 were significantly upregulated in COPD exosomes, while miR-155, miR-126 and miR-146a were notably downregulated. No significant differences were observed for miR-34a, miR-210, miR-199a-5p or miR-1246.

Table 2 summarizes the relative expression levels of the five differentially expressed sputum-derived exosomal miRNAs in COPD patients and healthy controls. COPD patients showed significant upregulation of miR-21 and miR-223 and downregulation of miR-155, miR-126, and miR-146a compared with controls. Specifically, miR-21 (fold change = 3.4; *p* < 0.001) and miR-223 (fold change = 2.1; *p* = 0.004) were increased, whereas miR-155 (fold change = 0.35; *p* = 0.002), miR-126 (fold change = 0.42; *p* = 0.009), and miR-146a (fold change = 0.28; *p* = 0.006) were decreased in COPD patients, with corresponding 95% confidence intervals shown in the table.

Moreover, analysis revealed a moderate positive correlation between CAT score and miR-21 levels in COPD patients (Pearson’s r = 0.445, *p* = 0.049), indicating that higher miR-21 expression is associated with increased symptom burden as assessed by the COPD Assessment Test.

**Figure 2:**
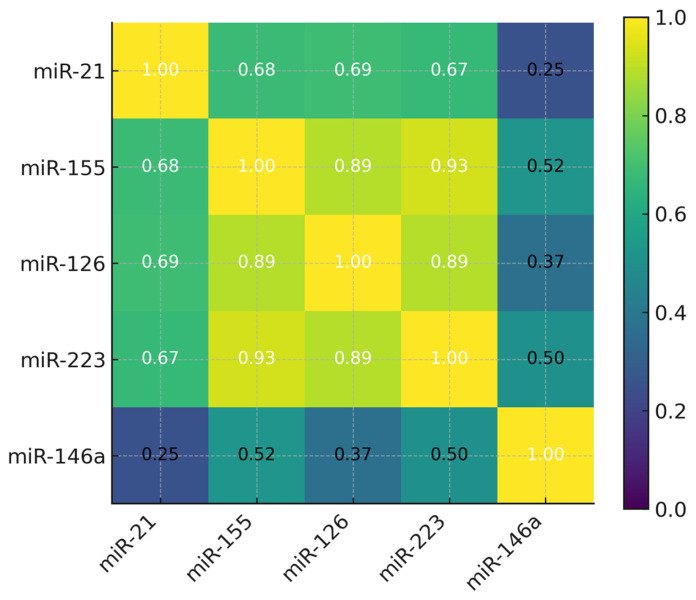
Correlation matrix of miRNAs expression levels.

Figure 2 shows the correlations between miR-155, miR-223, miR-146a, miR-126 and miR-21.

Figure 2 presents the correlation matrix of exosomal miRNA expression levels following log-transformation of normalized 2^−ΔCt^ values and systematic outlier screening. The strongest correlations were observed between miR-155 and miR-223 (r = 0.93, *p* < 0.001), miR-155 and miR-126 (r = 0.89, *p* < 0.001), and miR-126 and miR-223 (r = 0.89, *p* < 0.001), indicating that these miRNAs may be partially co-regulated and participate in related inflammatory pathways in COPD. miR-146a demonstrated only moderate correlations with the other miRNAs (r = 0.37–0.52), suggesting that it provides distinct biological information within the panel. miR-21 showed moderate correlations with miR-155, miR-126, and miR-223 (r = 0.67–0.69), and a weak association with miR-146a (r = 0.25), consistent with a more independent regulatory pattern. Importantly, none of the miRNA pairs exceeded r = 0.93, reflecting a heterogeneous correlation structure. This diversity suggests that the included miRNAs are not redundant and supports the use of multi-marker combinations to improve diagnostic discrimination in COPD.

### 3.4. Diagnostic Performance for COPD Detection

**Figure 3:**
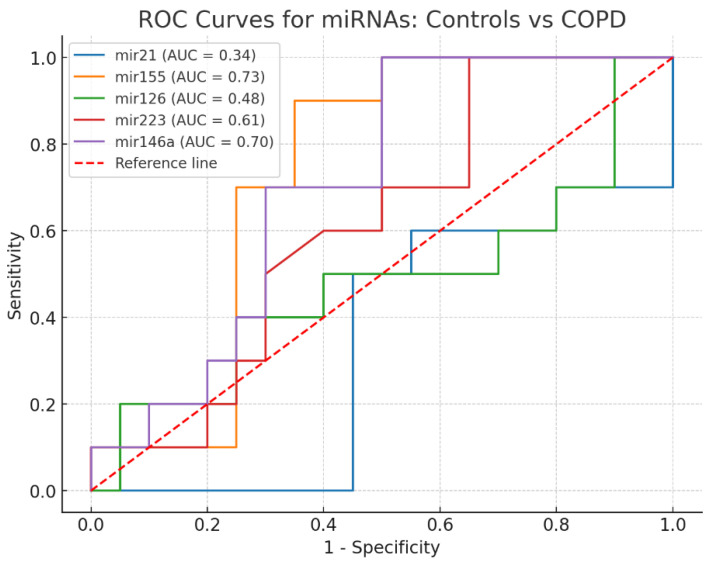
ROC curve for discriminating healthy controls from COPD patients using each individual miRNA. The red diagonal line represents the line of no discrimination (area under the curve = 0.5), indicating performance equivalent to chance.

ROC curve analysis was performed to evaluate the diagnostic performance of individual exosomal miRNAs for distinguishing COPD patients from healthy controls, as presented in Figure 3.

ROC curve analysis revealed that among individual miRNAs, miR-155 demonstrated the best diagnostic performance for distinguishing COPD patients from healthy controls (AUC = 0.730; sensitivity 80%; specificity 80%), followed by miR-146a (AUC = 0.700). miR-223, miR-21, and miR-126 showed lower discriminative ability (Table 3). All AUC values are now presented together with their 95% confidence intervals and *p*-values.

Although several miRNAs showed statistically significant AUCs compared with the null hypothesis of AUC = 0.5, pairwise comparisons of AUCs using the DeLong test did not identify any statistically significant differences between biomarkers, likely reflecting the limited sample size.

When miRNAs were combined using logistic regression models, their diagnostic accuracy improved substantially. The combination of miR-155 + miR-126 + miR-146a achieved an AUC of 0.841, with 90% sensitivity and 95% specificity, representing the best balance between true positive and true negative rates. Adding miR-223 to this triple panel provided only marginal improvement (AUC = 0.845) without further gains in sensitivity or specificity (Table 4).

These findings suggest that although certain single miRNAs, especially miR-155, show promise as biomarkers, a multi-marker approach provides better diagnostic accuracy. This could lead to more precise and earlier detection of COPD, while reducing the number of false positives. Such an approach is especially valuable in clinical settings where misclassification may lead to unnecessary treatment or missed diagnoses.

### 3.5. Diagnostic Performance for COPD Stage Discrimination

**Figure 4:**
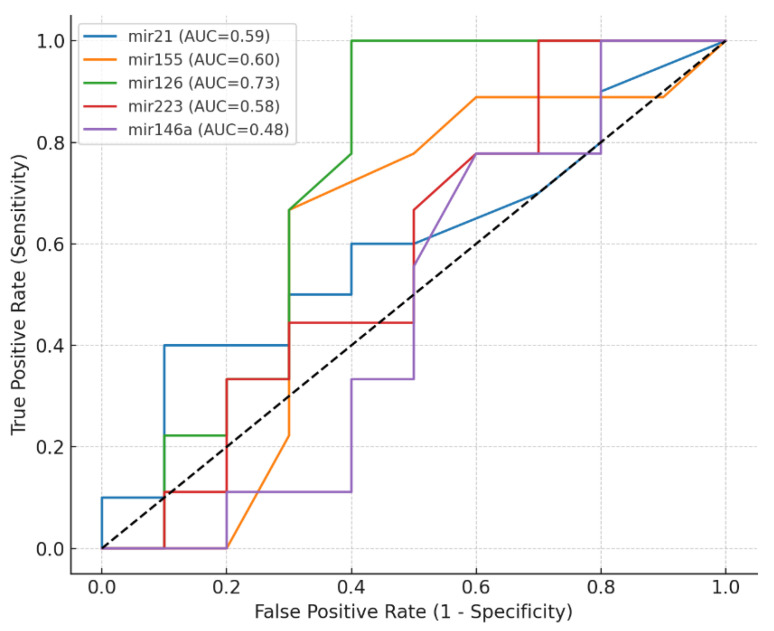
ROC Curves for miRNAs to discriminate mild COPD (GOLD stage I) from moderate/severe COPD (GOLD stages II–III). The black diagonal line represents the line of no discrimination (area under the curve = 0.5), indicating performance equivalent to chance.

The ROC analysis (Figure 4) demonstrates the diagnostic performance of five circulating miRNAs—miR-21, miR-155, miR-126, miR-223, and miR-146a—in distinguishing mild COPD (GOLD stage I) from moderate/severe COPD (GOLD stages II–III).

Among single biomarkers, miR-126 demonstrated the highest discriminative ability, with an AUC of 0.728, achieving 100% sensitivity and 60.0% specificity at the optimal cut-off of 0.32. This suggests that miR-126 can accurately identify all patients with mild COPD, albeit with moderate specificity. miR-155 (AUC = 0.600) showed balanced sensitivity (66.7%) and specificity (70.0%), while miR-21 (AUC = 0.595) exhibited high specificity (90.0%) but relatively low sensitivity (40.0%). In contrast, miR-223 (AUC = 0.583) and miR-146a (AUC = 0.478) had limited standalone diagnostic value (Table 5).

Evaluation of multi-marker combinations showed improved diagnostic performance compared to most single biomarkers (Table 6). The combination of miR-155, miR-126, and miR-146a achieved the highest overall accuracy (AUC = 0.778) with 66.7% sensitivity and 90.0% specificity at a probability threshold of 0.527. Another panel combining, miR-155, miR-223, and miR-146a, achieved the same AUC (0.778) but offered perfect specificity (100%) at the expense of lower sensitivity (55.6%). Two-marker combinations, such as miR-155 + miR-146a (AUC = 0.744; 55.6% sensitivity; 100% specificity), also demonstrated strong specificity, indicating minimal false-positive results.

Overall, these findings suggest that while miR-126 is the strongest single biomarker for COPD stage discrimination, combining selected miRNAs—particularly miR-155, miR-126, and miR-146a—can improve diagnostic precision, especially when high specificity is required for clinical decision-making.

## 4. Discussion

This study demonstrates that sputum-derived exosomal miRNAs exhibit distinct expression patterns in COPD, supporting their relevance as accessible biomarkers of airway pathology. We observed upregulation of miR-21 and miR-223, together with downregulation of miR-155, miR-126, and miR-146a in COPD compared with healthy controls. These alterations are broadly consistent with previously described inflammatory and structural pathways implicated in COPD and reinforce the utility of sputum exosomes as a biologically meaningful compartment for disease profiling [2,8,9,10,13].

Interpretation of these findings requires consideration of clinical differences between groups. Most COPD patients were former smokers, while the majority of controls were lifelong non-smokers, and COPD participants had a higher burden of comorbidities such as hypertension, hyperlipidemia, and depression. Although these factors do not directly alter sputum composition, both smoking exposure and systemic disease have been linked to changes in circulating miRNA profiles [2,10], and may influence airway-derived exosomal cargo. Furthermore, the impact of pharmacological therapies—including inhaled corticosteroids, long-acting bronchodilators, antihypertensives, lipid-lowering agents, and antidepressants—cannot be excluded, as several medication classes have been shown to modulate miRNA expression in epithelial, endothelial, and immune cells. The extent to which these factors contributed to our observed expression patterns cannot be determined in the current dataset.

The observed upregulation of miR-21 in sputum exosomes is in line with its established role as a regulator of PTEN/Akt/NF-κB signaling and its involvement in epithelial injury, fibroblast proliferation, and airway remodeling [9,19]. Our data agree with studies reporting elevated miR-21 in serum or airway samples from COPD patients [8,9,19], and extend these findings to sputum exosomes, supporting a local airway contribution to this dysregulation. The positive correlation between miR-21 levels and CAT score suggests that higher exosomal miR-21 may be associated with greater symptom burden, complementing prior work linking miR-21 to airflow limitation and disease progression [18,19]. However, the cross-sectional design precludes causal inference, and the functional impact of miR-21 in sputum exosomes remains to be directly tested in mechanistic studies.

Similarly, increased exosomal miR-223 in COPD sputum is consistent with its recognized role in neutrophilic inflammation and innate immune responses [20,21,22,23]. Elevated miR-223 has been described in lung tissue and BALF-derived exosomes from COPD patients, where it is associated with inflammatory cell recruitment and disease severity [18,21,22]. Our results agree with these reports and support the concept that miR-223 reflects activation of innate immune and inflammatory pathways in COPD across multiple biological compartments [20,21,22,23].

In contrast, miR-155 was downregulated in sputum exosomes from COPD patients, underscoring the compartment-specific complexity of this miRNA. While miR-155 is often elevated in asthma and autoimmune diseases, where it promotes Th1/Th17-driven inflammation [11,24], findings in COPD have been heterogeneous [10]. Tissue and BALF studies typically report increased miR-155 expression and support a pro-inflammatory role in emphysema and airway remodeling [9,10,11], whereas exosomal studies, including those by Sai et al. [12], have demonstrated reduced miR-155 levels in airway-derived vesicles from COPD patients. Our data are consistent with this exosome-focused pattern which is compatible with previous studies [9,10,11,12,24], suggesting roles in innate immunity, although our dataset cannot establish mechanistic effects.

The downregulation of miR-126 and miR-146a in sputum exosomes further supports disruption of regulatory networks involved in epithelial repair and inflammatory control. miR-126 is a key regulator of vascular integrity, angiogenesis, and epithelial homeostasis [14,15,24,25]. Reduced miR-126 expression has been reported in endothelial cells and lung tissue from smokers and COPD patients [14,26], where it is linked to impaired repair and increased DNA damage. Our finding of decreased exosomal miR-126 in sputum is consistent with this literature and suggests a potential airway-level correlate of these systemic and tissue changes, although causal links to comorbid vascular disease cannot be established from the present dataset [15]. miR-146a, a negative regulator of TLR/NF-κB signaling [16,24,25], was likewise reduced in COPD sputum exosomes, in agreement with previous reports in airway epithelium and fibroblasts [16,25]. Such reductions have been associated with exaggerated NF-κB activation and heightened inflammatory responses in experimental systems [16,25]; our observations are concordant with these mechanistic data but do not independently prove loss of regulatory control in vivo.

From a diagnostic viewpoint, our study supports the emerging concept that combinations of exosomal miRNAs provide more informative signatures than individual markers [2,5]. Although miR-155 showed the highest individual discriminative ability among the single miRNAs examined, multi-marker panels combining miR-155, miR-126, and miR-146a yielded higher sensitivity and specificity for COPD detection and staging, in line with previous work demonstrating superior performance of exosomal miRNA panels over single biomarkers [2,22]. This pattern is consistent with the notion that COPD is driven by interacting inflammatory, structural, and reparative processes, which are unlikely to be captured by a single molecular indicator.

The correlation analysis further supports a network-based view of miRNA regulation in COPD. After log-transformation of normalized 2^−ΔCt^ values and outlier screening, we observed moderate-to-strong correlations among miR-155, miR-126, and miR-223, suggesting partial co-regulation or involvement in related inflammatory pathways [10,20,23,24,25]. miR-146a showed only moderate associations with these miRNAs, consistent with its role as a feedback regulator rather than a primary driver of inflammatory amplification [16,24,25]. miR-21 displayed a distinct pattern, with moderate correlations to miR-126 and weaker relationships with the other miRNAs, in keeping with its recognized role in epithelial–mesenchymal transition and remodeling rather than neutrophilic or Th1/Th17-dominant inflammation [19,26,27,28]. Although these correlation structures cannot define causal interactions, they support the concept of partially overlapping but non-redundant regulatory axes—immune–inflammatory and structural-repair—that may both need to be sampled for optimal biomarker performance [2,10,20].

The detection of exosomal miRNAs in sputum offers a minimally invasive and clinically feasible approach that could be integrated into specialized respiratory clinics with relatively minor adaptations of existing sputum induction protocols [5,13]. Exosomal miRNAs are stable, accessible, and mechanistically linked to disease pathways [3,4,5,6,7], making them attractive candidates for early diagnosis, risk stratification, and monitoring treatment response in COPD [2,5,8,22]. In the longer term, dynamic changes in sputum exosomal miRNA profiles may prove useful for predicting exacerbation risk or tracking therapeutic responses, and deregulated miRNAs such as miR-21, miR-126, miR-146a, and miR-223 could represent potential targets for RNA-based interventions [8,9,15,16,18,19,20,26,27,28]. These applications, however, will require validation in larger, longitudinal, and mechanistically oriented studies.

Because our study focused solely on sputum-derived exosomal miRNAs, we were not able to determine whether the observed expression patterns are specific to the bronchial compartment or reflect broader systemic dysregulation. Serum samples were not collected; therefore, direct comparisons between airway and circulating miRNA levels could not be made. Previous studies have shown that several of these miRNAs, including miR-21 and miR-223, may also be elevated in peripheral blood of COPD patients [8,19], suggesting that both local and systemic inflammatory processes may influence their levels. Future studies incorporating matched sputum–serum sampling will be necessary to clarify the compartment-specific versus systemic contribution to these miRNA signatures.

Future research could also focus on mapping miRNA–transcription factor (TF) co-regulatory networks. Exploratory analysis using the TransmiR v3.0 database [28] suggested enrichment of several TFs (including NR4A1, TGFB1, IL1B, LMO3, ZBTB16, ETS2, AP-1, FOXP3, PML, LDB1) potentially regulating the differentially expressed sputum-derived exosomal miRNAs identified here. Although beyond the scope of the present work, systematic characterization of such miRNA–TF networks may help elucidate upstream signals that drive COPD-related miRNA dysregulation and could identify additional diagnostic or therapeutic nodes [10,18,28].

This study has several important limitations. First, the sample size was small and exploratory, which reduces statistical power, increases uncertainty around diagnostic estimates, and limits our ability to adjust for comorbidities or medication use. The limited number of participants also underpowers ROC analyses and pairwise AUC comparisons, making the diagnostic performance estimates susceptible to overfitting and optimistic bias. Second, controls included both never-smokers and former light smokers, whereas most COPD participants were heavy former smokers; given the well-established effects of cigarette smoke on exosomal miRNA expression, residual confounding by cumulative smoking exposure remains likely. Third, COPD participants had higher rates of comorbidities such as hypertension, hyperlipidemia, and depression, and the influence of these conditions—and their pharmacological treatments—cannot be ruled out, as many medications are known to modulate miRNA expression. Fourth, systemic and sputum inflammatory biomarkers (e.g., CRP, leukocyte counts, sputum cell differentials) were not collected, limiting our ability to relate exosomal miRNA levels to objective measures of inflammation; therefore, inflammation-related mechanistic interpretations should be considered hypothesis-generating. Fifth, serum miRNA levels were not assessed, preventing evaluation of compartment specificity and whether the observed miRNA alterations reflect airway-localized or systemic processes. Sixth, although RNA purity was confirmed, RNA integrity was not evaluated, no exogenous spike-in controls were used, and normalization relied on U6 snRNA, which is nuclear and not exosome-derived, potentially introducing normalization bias. Finally, diagnostic performance was calculated in the same cohort where biomarkers were identified, without an independent validation set; thus, the reported AUC values—tested under the null hypothesis that AUC = 0.5 using two-sided DeLong comparisons—should be interpreted as exploratory until validated in external cohorts.

## 5. Conclusions

This study demonstrates that sputum-derived exosomal miRNAs exhibit a disease-specific signature in COPD, characterized by the upregulation of miR-21 and miR-223, and the downregulation of miR-155, miR-126, and miR-146a. These alterations align with key pathogenic processes, including airway inflammation, impaired epithelial repair, vascular dysfunction, and defective immune regulation. While individual miRNAs such as miR-155 showed moderate diagnostic potential, multi-marker panels combining miR-155, miR-126, and miR-146a achieved superior sensitivity and specificity, underscoring the value of composite biomarker strategies. The compartment-specific downregulation of miR-155 and miR-146a in sputum exosomes highlights the importance of sample type in miRNA profiling and may reflect unique airway-specific mechanisms that contribute to infection susceptibility and persistent inflammation in COPD. Overall, our findings support sputum exosomal miRNAs as promising, minimally invasive biomarkers with potential applications in early diagnosis, disease staging, and patient stratification, while also providing novel insights into the molecular underpinnings of COPD. Larger longitudinal studies and mechanistic experiments are now warranted to validate these observations and explore their therapeutic implications.

## Figures and Tables

**Table 1:** COPD patients (20) and Healthy Controls (10) demographic and clinical characteristics.

Parameters	COPD Patients (*n* = 20)	Controls (*n* = 10)	*p*-Value
Gender			
Males, n (%)	19 (95)	9 (90)	0.569
Females, n (%)	1 (5)	1 (10)	
Age (years)	70 ± 8	65 ± 5	0.342
BMI (Kg/m^2^)	31 ± 6	28 ± 7	0.423
Comorbidities, yes, n (%)			
Arterial Hypertension yes, n (%)	18 (90)	5 (50)	0.140
Hyperlipidemia yes, n (%)	15 (70)	5 (50)	0.240
Diabetes mellitus (%)	4 (20%)	1 (10%)	0.640
Coronary artery disease (%)	3 (15%)	1 (10%)	0.990
Obstructive sleep apnea yes, n (%)	10 (50)	3 (30)	0.321
Depression yes, n (%)	9 (45)	2 (20)	0.150
Smoking status			
Former smokers	18 (90)	5 (50)	<0.001
Never smokers	2 (10)	5 (50)	0.100
Pack-years	77 ± 42	8 ± 2	0.001
FEV_1_/FVC	66 ± 5	76 ± 2	0.001
FEV_1_ (L)	1.95 ± 0.68	3.05 ± 0.52	<0.001
FEV_1_ (%)	72 ± 8	110 ± 2	0.001
GOLD stage		-	-
Stage I, n (%)	10 (50)		
Stage II, n (%)	9 (45)		
Stage III, n (%)	1(5)		
Stage IV, n (%)	0 (0)		
CAT score	16 ± 9	-	-
Εxacerbation the last year, yes, n (%)	6 (30)	-	-
Hospitalization the last year, yes, n (%)	6 (30)	-	-

Abbreviations: BMI, body mass index; CAT, COPD Assessment Test; FEV_1_, Forced expiratory volume in the first second; FVC, Forced Vital Capacity.

**Table 2:** Relative expression of sputum-derived exosomal miRNAs in COPD patients and healthy controls, with fold changes and 95% confidence intervals.

miRNA	COPD Patients (Mean ± SD)	Controls (Mean ± SD)	Fold Change (COPD vs. Controls)	95% CI for Expression (COPD vs. Controls) *	*p*-Value
miR-21	0.3795 ± 0.5783	0.2017 ± 0.03061	3.4	0.12–0.64 vs. 0.18–0.22	<0.001
miR-155	0.5500 ± 0.2572	1.040 ± 0.2875	0.35	0.43–0.67 vs. 0.86–1.22	0.002
miR-126	0.3467 ± 0.09689	1.114 ± 0.5377	0.42	0.30–0.39 vs. 0.80–1.42	0.009
miR-223	1.894 ± 4.445	1.040 ± 0.2802	2.1	0.00–3.79 vs. 0.86–1.22	0.004
miR-146a	0.8018 ± 0.6598	1.107 ± 0.4374	0.28	0.49–1.12 vs. 0.87–1.35	0.006

* 95% confidence intervals (CI) refer to the relative expression ranges in COPD patients and controls used to derive the reported fold changes.

**Table 3:** Diagnostic performance of individual exosomal miRNAs for discriminating COPD patients from healthy controls. All AUC values in this table are reported together with their 95% confidence intervals and *p*-values for all AUC estimates.

Biomarker(s)	AUC	95% CI	*p*-Value	Optimal Cut-Off (Probability)	Sensitivity (%)	Specificity (%)
miR-21	0.340	0.14–0.54	0.321	0.431	55	60
miR-155	0.730	0.53–0.93	0.012	0.318	80	80
miR-126	0.480	0.26–0.70	0.790	0.385	65	70
miR-223	0.610	0.39–0.82	0.208	0.412	60	70
miR-146a	0.700	0.49–0.91	0.030	0.345	75	75

**Table 4:** Diagnostic performance of combined exosomal miRNAs for discriminating COPD patients from healthy controls. All AUC values are presented with their 95% confidence intervals and *p*-values for all AUC estimates.

Biomarker(s)	AUC	95% CI	*p*-Value	Sensitivity (%)	Specificity (%)
miR-155 + miR-126	0.805	0.65–0.96	0.004	85	85
miR-155 + miR-146a	0.812	0.66–0.97	0.003	85	90
miR-155 + miR-126 + miR-146a	0.841	0.69–0.98	0.001	90	95
miR-155 + miR-126 + miR-146a + miR-223	0.845	0.71–0.98	0.001	90	95

**Table 5:** Diagnostic performance of individual exosomal miRNAs for discriminating mild COPD (GOLD stage I) from moderate/severe COPD (GOLD stages II–III). All AUC values include 95% confidence intervals and *p*-values for all AUC estimates.

miRNA	AUC	95% CI	*p*-Value	Optimal Cut-Off	Sensitivity	Specificity
miR-21	0.595	0.38–0.81	0.320	0.45	40.0%	90.0%
miR-155	0.600	0.38–0.82	0.298	0.60	66.7%	70.0%
miR-126	0.728	0.50–0.96	0.028	0.32	100%	60.0%
miR-223	0.583	0.36–0.80	0.378	0.29	100%	30.0%
miR-146a	0.478	0.25–0.71	0.790	0.24	100%	20.0%

**Table 6:** Diagnostic performance of combined exosomal miRNAs for discriminating mild COPD (GOLD stage I) from moderate/severe COPD (GOLD stages II–III). All AUC values are presented with their 95% confidence intervals and *p*-values for all AUC estimates.

Biomarker(s)	AUC	95%CI	*p*-Value	Sensitivity (%)	Specificity (%)
miR-155 + miR-223 + miR-146a	0.778	0.57–0.99	0.016	55.6	100
miR-155 + miR-146a	0.744	0.52–0.96	0.031	55.6	100
miR-155 + miR-126 + miR-146a	0.778	0.57–0.99	0.016	66.7	90

## Data Availability

The original contributions presented in this study are included in the article/Supplementary Materials. Further inquiries can be directed to the corresponding author.

## Supplementary Materials

The following supporting information can be downloaded at: https://www.mdpi.com/article/10.3390/biomedicines13123027/s1, Figure S1: (A) Representative transmission electron microscopy (TEM) image of sputum-derived extracellular vesicles (EVs) from a healthy control, demonstrating round, membrane-bound vesicles with typical exosomal morphology (scale bar: 200 nm). (B) TEM image of sputum-derived EVs from a COPD patient, showing similar vesicular structures with diameters up to ~200 nm (scale bar: 200 nm). (C) Western blot analysis of exosomes isolated from sputum samples of healthy controls (HC) and COPD patients. Exosomal identity was confirmed by the presence of the tetraspanin markers CD81 and CD9, while the absence of calnexin verified minimal contamination with non-exosomal cellular components. Panels A–C adapted from [13].
