# Supplementary figures and images for: Sputum Exosomal microRNAs as Non-Invasive Biomarkers in COPD: A Cross-Sectional Study
Source: Biomedicines. 2025 Dec 10;13(12):3027. doi: 10.3390/biomedicines13123027 (PMC12730506; doi:10.3390/biomedicines13123027)

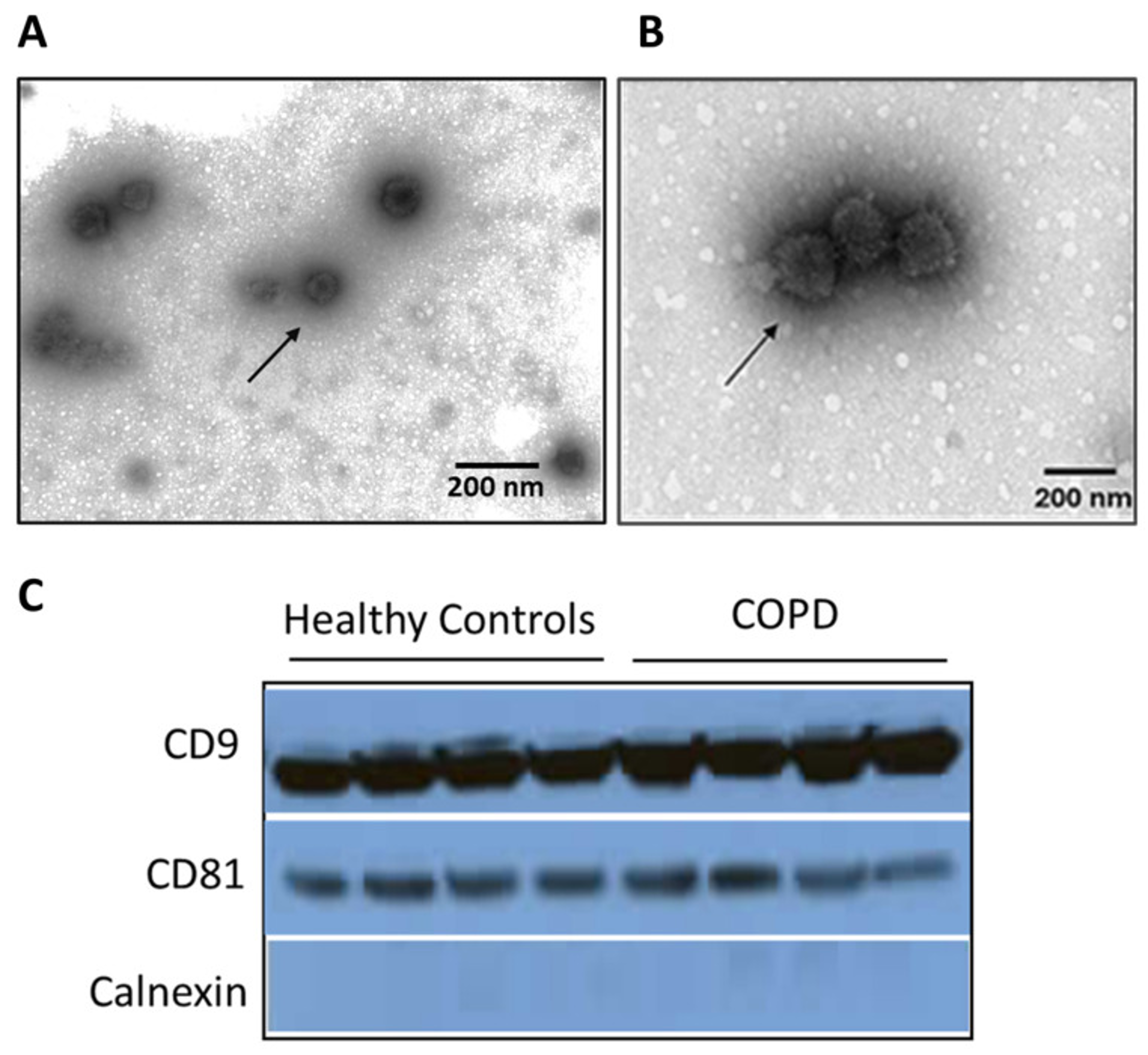

Supplement: Supplementary file 1 [file biomedicines-13-03027-s001.zip › biomedicines-3964784-Figure S1.tif]
